# Thin endometrium is associated with higher risks of preterm birth and low birth weight after frozen single blastocyst transfer

**DOI:** 10.3389/fendo.2022.1040140

**Published:** 2022-11-10

**Authors:** Yu Zheng, Biao Chen, Jun Dai, Bei Xu, Jihui Ai, Lei Jin, Xiyuan Dong

**Affiliations:** ^1^ Reproductive Medicine Center, Tongji Hospital, Tongji Medical College, Huazhong University of Science and Technology, Wuhan, China; ^2^ Department of Obstetrics and Gynecology, Tongji Hospital, Tongji Medical College, Huazhong University of Science and Technology, Wuhan, China

**Keywords:** endometrial thickness, preterm birth, low birth weight, miscarriage, frozen embryo transfer

## Abstract

**Background:**

It has been demonstrated that a thin endometrium is associated with a lower chance of pregnancy, but there is a paucity of research into whether a thin endometrium adversely affects perinatal outcomes.

**Methods:**

This was a retrospective cohort study on 10098 frozen cycles with single blastocyst transfer, resulting in 5505 singleton clinical pregnancies, and 4314 singleton live births. Patients were divided into a thin endometrium group (<8 mm) and a normal endometrium group (≥8 mm). Multivariable logistic regression with restricted cubic splines, receiver operating characteristic curve, and multivariable linear model were used for statistical analysis.

**Results:**

The incidences of preterm birth (15.65 vs. 9.80%, aOR=1.69 [1.19-2.42]), low birth weight (8.40 vs. 4.10%, aOR=2.05 [1.27-3.30]) and gestational diabetes (6.87 vs. 4.17%, aOR=1.74 [1.05-2.90]) were all higher in the endometrial thickness (EMT) <8 mm group. The miscarriage rate was higher in the EMT <8 mm group than the EMT ≥8 mm group (27.91 vs. 20.39%, aOR=1.40 [1.10-1.79]).

**Conclusion:**

A thin endometrium may be associated with a higher incidence of preterm birth, low birth weight, and miscarriage. Therefore, embryo transfer should be performed with caution in these patients, and postponing to a later cycle with a thicker endometrium should be considered.

## Introduction

A receptive endometrium and competent embryos are two well-established determinants of achieving a pregnancy in both natural and assisted reproductive technology (ART) conceptions (1). Over recent decades, many improvements have been made in embryo culture; however, identifying the receptivity of the endometrium remains a challenge. Conventional endometrial receptive markers, such as histological changes and ultrasound characteristics, show poor ability to predict clinical pregnancy events (2).

Trans-vaginal ultrasound is a non-invasive and effective tool for assessing the endometrium (3). Endometrial thickness (EMT) is measured in the mid-sagittal plane by ultrasound, and is routinely used to evaluate whether the endometrium has reached a receptive status. However, previous studies assessing the association between EMT and ART success rates have not drawn conclusive results. Some studies found decreased rates of both pregnancy and live birth in cycles with a low EMT (4–6); however, other studies show no significant effect of EMT on pregnancy outcomes (7–9). Two meta-analyses show that clinical pregnancy rate declines as EMT decreases, but that EMT is a poor predictor of pregnancy with limited sensitivity and specificity (2, 10). It is noteworthy that neither study accounted for the effect of EMT on miscarriage and live birth.

It is known that the risks of preterm birth (PTB) and low birth weight (LBW) are higher for ART births as compared with natural births (11). However, there is a paucity of evidence on perinatal outcomes in women with thin endometria during ART cycles. Several studies show that a low EMT (<7-8 mm) is associated with higher incidences of PTB, small-for-gestational-age (SGA), and LBW (12–16). Additionally, there is concern that a thin endometrial lining might not be able to support proper development of the placenta or fetus, and thus lead to adverse perinatal outcomes.

For patients with thin endometria, it is important to predict their ART success and safety. In a cycle with thin endometrium, it is often a dilemma to decide whether embryo transfer should be continued. Clinical evidence is needed to address these issues, and therefore we performed the current study. We hypothesized that a thin endometrium would result in adverse perinatal outcomes; and the object of this study was to assess the effect of EMT on maternal and neonatal outcomes.

## Materials and methods

### Study patients

We performed a retrospective cohort study on patients who underwent embryo transfer at our center between January 2016 and December 2018. The inclusion criteria were: 1) frozen-thawed cycles; 2) single blastocyst transfer; 3) Chinese population. The following cycles were excluded: 1) monozygotic twin pregnancies; 2) donation or frozen oocytes/sperm; 3) preimplantation genetic testing (PGT); 4) uterine malformations, and uterine fibroids/adenomyomas which distorted the uterine cavity. Patients with small single fibroids/adenomyomas <30 mm in diameter were not excluded because a previous study has shown them to have no significant adverse effect on IVF outcomes (17); 5) patients with hypertension disorders, diabetes mellitus, or immune disorders; 6) patients with intrauterine adhesions (IUA), who had abnormal uterine morphology after treatment with hysteroscopic adhesionolysis. We performed a routine hysteroscopy examination for patients with a history of IUA, and administered post-operative oral estrogen. Those patients with a general normal uterine morphology after treatment were not excluded; 7) patients with missing data. Finally, a total of 10098 cycles, resulting in 5505 singleton clinical pregnancies, and 4314 singleton live births were enrolled for the final analysis.

Endometrial thickness was measured on the day of administration with progesterone by three experienced sonographers during the study period. The cycles were divided into a thin endometrium group (EMT <8 mm) and a normal endometrium group (EMT ≥8 mm), based on the target EMT at our center being ≥8 mm. This cut-off has also been used in many recently published studies (2, 14, 15). This current study was performed with the approval of the Institutional Review Board (IRB) of Tongji Hospital. All included patients gave written consent regarding the use of their data. The data were fully anonymized before analysis.

### Endometrial preparation

Endometrial preparation was performed using a natural cycle (NC) protocol, an artificial cycle (AC) protocol, or a downregulation combined with AC (DR+AC) protocol. Regarding the NC protocol, serial trans-vaginal ultrasound scans were performed until the endometrial thickness reached ≥8 mm or approximated the level in the stimulated cycle. The timing of ovulation was estimated by a combined analysis of ultrasound results, the LH level and the P level. Regarding the AC protocol, E2 valerate tablets (PROGYNOVA, Bayer, Germany) were administered at 2 mg/d on day 2-4, 4 mg/d on day 5-7, and 6 mg/d on day 8-11. Serial ultrasound scans were performed from day 11-12. If EMT was <8 mm, the dosage of E2 was increased to 8 mg/d for 3 d. If EMT was still <8 mm, E2 with 8 mg/d was used for 3 more days. The maximal dosage of E2 was 8mg/d for 6 d. When the endometrial thickness reached ≥8 mm or approximated the level in the stimulated cycle, 40 mg intramuscular P (P injection, Xianju, China) and 20mg oral dydrogesterone (Duphaston, Abbott, Netherlands) were used to transform the endometrium. Regarding the DR+AC protocol, a depot GnRH-a (leuprorelin acetate [BEIYI, Lizhu, China]) of 3.75 mg was subcutaneously administered on the second day of menstruation. Oral estrogen was administrated on the 28th day after leuprorelin injection. The following course was similar to the AC protocol.

### Embryo vitrification, warming and transfer

Embryo vitrification and warming were performed as previously described (18). The embryos were vitrified within 2 h of scoring. The entire vitrification procedure was performed at room temperature (22-25°C). Embryos were equilibrated in equilibration solution (ES; Vitrification kit, Kitazato, Japan), containing 7.5% ethylene glycol and 7.5% dimethylsulfoxide (DMSO), for 5-10 min. The embryos were then transferred into vitrification solution (VS; Vitrification kit, Kitazato, Japan), which contained 15% ethylene glycol, 15% DMSO, and 0.5 mol/L sucrose; they were subsequently loaded onto the surface of a Cryotop System (Kitazato, Japan) within 40-60 s and then immediately submerged in liquid nitrogen.

On the day of transfer, embryos were warmed at room temperature (22–25°C). They were transferred to thawing solution (TS; Vitrification kit, Kitazato, Japan), which contained 1.0 mol/L sucrose, for 1 min, followed by 3 min in diluent solution (DS; Vitrification kit, Kitazato, Japan), which contained 0.5 mol/L sucrose. They were then washed twice in washing solution 1 and 2 (WS1 and WS2; Vitrification kit, Kitazato, Japan) for 5 min each. The warmed embryos were then cultured for at least 2 h before post-warming evaluation. The temperature of the TS, WS2, and culture media were maintained at 37°C. After warming, the embryos were checked for survival under an inverted microscope. They were immediately transferred after post-warming evaluation.

Blastocysts were transferred 5 d after P transformation. Luteal phase support was provided from the day of transfer until the 10th week of gestation, with 90 mg/d vaginal P (8% Crinone, Merck, UK) and 20mg/d oral dydrogesterone (Duphaston, Abbott, Netherlands).

### Outcome measures

The primary outcomes were PTB and LBW. Other perinatal metrics and cycle outcomes were also analyzed. A clinical pregnancy was diagnosed when serum hCG level reached >20 IU/l at 2 weeks after transfer and a gestational sac was detected on ultrasound at 5–7 weeks after transfer. A live birth was defined as complete expulsion or extraction of a live baby after the 28th week of gestation. Maternal outcomes included gestational age, delivery mode, PTB (<37 weeks of gestation), very preterm birth (VPTB, <32 weeks of gestation) and maternal complications. Maternal complications included gestational hypertension disorders (International Classification of Diseases (ICD) 10 codes O13-15), gestational diabetes mellitus (O24), premature abruption of membrane (O42), placenta previa (O44), placenta abruption (O45), and placenta accrete (O73). Neonatal outcomes included birth weight, LBW (<2500 g), very low birth weight (VLBW, <1500 g), macrosomia (≥4000 g), SGA (<10th percentile of the average birth weight at the same gestational week), large-for-gestational age (LGA, >90th percentile of the average birth weight at the same gestational week), sex of newborn, congenital anomaly, pediatric intensive care unit (PICU) admission, and neonatal mortality.

### Statistical analysis

SAS 9.2 (SAS Inc., NC, USA) was used for statistical analysis. Continuous variables are shown as mean ± SD. Categorical variables are shown as number (percentage). Analysis of variance (ANOVA) and chi-square test were performed as appropriate. A P value <0.05 was considered statistically significant.

Univariable analysis was performed to identify the significant effectors of live birth (**Table S1**). Association analyses were performed using both means and cut-offs of EMT. EMT was firstly set as a continuous variable. Multivariable logistic regression and restricted cubic splines were used to analyze the effect of EMT on live birth (**Table S2**). ROC curves were used to assess the accuracy, sensitivity and specificity of EMT on predicting live birth. EMT was then set as a categorial variable, and multivariable logistic regression was used to analyze the associations between EMT and categorical outcomes (such as live birth, PTB and LBW) with age, BMI, type of infertility, duration of infertility, IVF indication, the number of oocytes retrieved, the day of blastocyst development, and blastocyst scores as covariates (**Tables S3**, **S4**). Multivariable general linear models were used to evaluate the associations between EMT and continuous outcomes (such as gestational age and birth weight), with the same covariates as in the logistic regression models.

## Results

**Figure 1:**
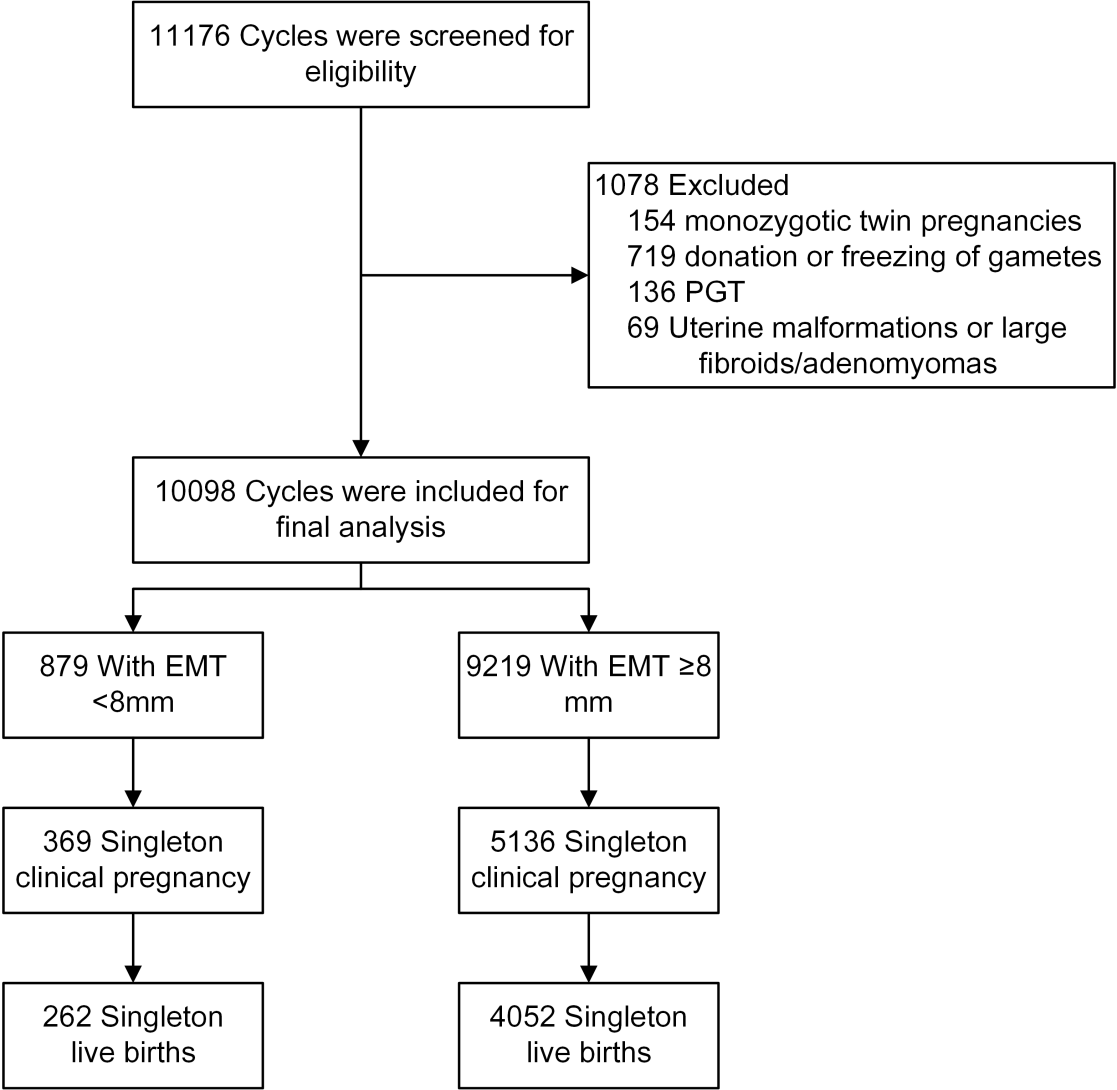
The flow chart of patient selection. EMT, endometrial thickness; PGT, preimplantation genetic testing.

There were 10098 frozen cycles with single blastocyst transfer, resulting in 5505 singleton clinical pregnancies, and 4314 singleton live births (**Figure 1**). The demographic and clinical features are shown in **Table 1**. Women with an EMT of <8 mm and ≥8 mm had a similar BMI. The mean age (33.21 ± 4.85 vs. 31.89 ± 4.71 y, P<0.001), the percentage of parity (24.57 vs. 16.51%, P<0.001), and the percentage of women with a history of PTB (2.73 vs. 1.30, P=0.001) were all higher, while the proportion of primary infertility (37.32 vs. 41.79%, P=0.010), the duration of infertility (3.31 ± 2.77 vs. 3.56 ± 2.82 y, P=0.015), serum AMH level (4.26 ± 3.24 vs. 5.30 ± 3.85 ng/ml, P<0.001), and AFC (10.70 ± 5.46 vs. 12.70 ± 6.03, P<0.001) were all lower in the EMT <8 mm group, compared with those in EMT ≥8 mm. A significant difference was also found in infertility cause between the two groups. Women with an EMT of <8 mm had a lower number of oocytes retrieved (9.44 ± 4.52 vs. 11.08 ± 4.75, P<0.001). Significant differences were also found in fertilization method and endometrial preparation protocols, but the proportion of women with a history of miscarriage, the number of available embryos, the day of embryo development, and the grade of embryo transferred were all comparable between the two groups.

**Table 1:** Demographic and clinical features according to EMT.

	EMT <8 mm	EMT ≥8 mm	P value
No. of cycles	879	9219	
Age (year)	33.21 ± 4.85	31.89 ± 4.71	<0.001
BMI (kg/m^2^)	21.65 ± 2.99	21.82 ± 2.98	0.140
Primary infertility, n (%)	328 (37.32)	3853 (41.79)	0.010
Duration of infertility (year)	3.31 ± 2.77	3.56 ± 2.82	0.015
Infertility cause, n (%)			<0.001
Tubal factors	343 (39.02)	3360 (36.45)	
Endometriosis	65 (7.39)	623 (6.76)	
Male factors	49 (5.57)	951 (10.32)	
Ovulation disorders	178 (20.25)	1921 (20.84)	
Unexplained factor	14 (1.59)	333 (3.61)	
Diminished ovarian reserve	199 (22.64)	1769 (19.19)	
Others	31 (3.53)	262 (2.84)	
AMH (ng/ml)	4.26 ± 3.24	5.30 ± 3.85	<0.001
AFC	10.70 ± 5.46	12.70 ± 6.03	<0.001
No. of oocytes	9.44 ± 4.52	11.08 ± 4.75	<0.001
Fertilization method, n (%)			<0.001
IVF	681 (77.47)	6489 (70.39)	
ICSI	198 (22.53)	2730 (29.61)	
No. of available embryos	3.95 ± 2.56	4.04 ± 2.72	0.392
Endometrial preparation, n (%)			<0.001
Natural cycle	54 (6.14)	508 (5.51)	
Artificial cycle	699 (79.52)	7828 (84.91)	
Down-regulation + artificial cycle	126 (14.33)	883 (9.58)	
Day of embryo development, n (%)			0.907
Day 5	469 (53.36)	4900 (53.15)	
Day 6	410 (46.64)	4319 (46.85)	
Grade of embryo transferred, n (%)			0.660
Good	78 (8.87)	906 (9.83)	
Fair	519 (59.04)	5380 (58.36)	
Poor	282 (32.08)	2933 (31.81)	

Maternal outcomes are presented in **Table 2**. The mean gestational age was slightly but statistically lower in the EMT <8 mm group compared with EMT ≥8 mm (38.28 ± 1.86 vs. 38.58 ± 1.68 weeks, SMD=-0.31 [-0.52 ~ -0.10]). The incidences of PTB (15.65 vs. 9.80%, aOR=1.69 [1.19-2.42]) and gestational diabetes (6.87 vs. 4.17%, aOR=1.74 [1.05-2.90]) were all higher in the EMT <8 mm group. There was no difference in delivery mode, VPTB, placenta previa, placenta abruption, premature abruption of membranes, oligohydramnios, or polyhydramnios between the two groups. Incidences of hypertension disorders and placenta accrete/increta were higher in the EMT <8 mm group.

**Table 2:** Maternal outcomes as a function of EMT.

	EMT <8 mm	EMT ≥8 mm	P value
No. of deliveries	262	4052	
Gestational age (week)	38.28 ± 1.86	38.58 ± 1.68	0.005
SMD^b^ (95% Cl)	-0.31 (-0.52 ~ -0.10)	REF	0.009
Delivery mode, n (%)			0.349
Cesarean section	236 (90.08)	3572 (88.15)	
Vaginal delivery	26 (9.92)	480 (11.85)	
PTB, n (%)	41 (15.65)	397 (9.80)	0.002
OR^a^ (95% Cl)	1.69 (1.19-2.42)	REF	0.004
VPTB, n (%)	4 (1.53)	47 (1.16)	0.595
OR^a^ (95% Cl)	1.48 (0.53-4.18)	REF	0.457
Gestational diabetes, n (%)	18 (6.87)	169 (4.17)	0.038
OR^a^ (95% Cl)	1.74 (1.05-2.90)	REF	0.033
Hypertension, n (%)	12 (4.58)	104 (2.57)	0.051
OR^a^ (95% Cl)	1.95 (1.05-3.62)	REF	0.035
Placenta previa, n (%)	10 (3.82)	120 (2.96)	0.433
OR^a^ (95% Cl)	1.31 (0.68-2.55)	REF	0.421
Placenta abruption, n (%)	0 (0.00)	4 (0.10)	–
OR^a^ (95% Cl)	NA	NA	
Placenta accreta/increta, n (%)	5 (1.91)	21 (0.52)	0.005
OR^a^ (95% Cl)	3.74 (1.38-10.17)	REF	0.010
PROM, n (%)	2 (0.76)	22 (0.54)	0.642
OR^a^ (95% Cl)	1.42 (0.33-6.14)	REF	0.636
Oligohydramnios, n (%)	0 (0.00)	8 (0.20)	–
OR^a^ (95% Cl)	NA	NA	
Polyhydramnios, n (%)	1 (0.38)	4 (0.10)	0.192
OR^a^ (95% Cl)	3.32 (0.33-33.15)	REF	0.307

a adjustment with multivariable logistic regression model.

b adjustment with multivariable general linear model; SMD, standardized mean difference; OR, odds ratio; PTB, preterm birth; VPTB, very preterm birth; PROM, premature rupture of membrane; REF, reference; NA, not applicable.

**Table 3** shows the neonatal outcomes. The mean birth weight was lower in the EMT <8 mm group as compared to EMT ≥8 mm (3217.12 ± 510.15 vs. 3366.21 ± 507.17 g, SMD=-148.52 [-212.99 ~ -84.04]). The incidence of LBW (8.40 vs. 4.10%, aOR=2.05 [1.27-3.30]) was higher, while the incidences of macrosomia (4.58 vs. 9.55%, aOR=0.49 [0.27-0.88]) and LGA (9.92 vs. 17.99%, aOR=0.51 [0.33-0.78]) were lower in the EMT <8 mm group. No differences were found in newborn sex, VLBW, SGA, congenital anomaly, PICU admission, or perinatal death between the two groups.

**Table 3:** Neonatal outcomes as a function of EMT.

	EMT <8 mm	EMT ≥8 mm	P value
No. of neonates	262	4052	
Birth weight (g)	3217.12 ± 510.15	3366.21 ± 507.17	<0.001
SMD^b^ (95% CI)	-148.52 (-212.99 ~ -84.04)	REF	<0.001
Sex of newborn, n (%)			0.179
Male	138 (52.57)	2305 (56.88)	
Female	124 (47.43)	1747 (43.12)	
LBW, n (%)	22 (8.40)	166 (4.10)	0.001
OR^a^ (95% CI)	2.05 (1.27-3.30)	REF	0.003
VLBW, n (%)	1 (0.38)	26 (0.64)	0.605
OR^a^ (95% CI)	0.66 (0.09-4.88)	REF	0.679
SGA, n (%)	16 (6.11)	212 (5.23)	0.540
OR^a^ (95% CI)	1.22 (0.72-2.06)	REF	0.471
LGA, n (%)	26 (9.92)	729 (17.99)	0.001
OR^a^ (95% CI)	0.51 (0.33-0.78)	REF	0.002
Macrosomia, n (%) OR^a^ (95% CI)	12 (4.58) 0.49 (0.27-0.88)	387 (9.55) REF	0.007 0.018
Congenital anomaly, n (%)	2 (0.76)	24 (0.59)	0.729
OR^a^ (95% CI)	1.24 (0.29-5.39)	REF	0.776
PICU admission, n (%)	1 (0.38)	7 (0.17)	0.446
OR^a^ (95% CI)	2.15 (0.26-17.36)	REF	0.478
Perinatal death, n (%)	1 (0.38)	7 (0.17)	0.446
OR^a^ (95% CI)	2.39 (0.29-19.86)	REF	0.420

a adjustment with multivariable logistic regression model.

b adjustment with multivariable general linear model; SMD, standardized mean difference; OR, odds ratio; LBW, low birth weight; VLBW, very low birth weight; SGA, small-for-gestational-age; LGA, large-for-gestational-age; PICU, pediatric intensive care unit; REF, reference; NA, not applicable.

**Figure S1** shows the results of association analysis of EMT and clinical pregnancy/live birth. A and B show the odds for clinical pregnancy and live birth with EMT as a continuous variable. Restricted cubic spline curves showed that the chances of clinical pregnancy and live birth both increased with increasing EMT. C and D display the predicting capacity of EMT on clinical pregnancy and live birth. ROC curves indicated that EMT was a poor predictor for clinical pregnancy or live birth, with areas under the curves (AUC) of 0.55 (0.53-0.56) and 0.55 (0.54-0.56), respectively. E and F are forest plots of the associations between EMT and clinical pregnancy and live birth. The results indicated that an EMT of 8 mm was a cut-off point, above which both the clinical pregnancy rate (CPR) and live birth rate (LBR) were significantly higher.

**Table S2** shows the cycle outcomes with EMT as a dichotomized variable with the cut-off at 8 mm. Compared with patients with an EMT of ≥8 mm, those with an EMT <8 mm had a lower CPR (41.98 vs. 55.71%, aOR=0.60 [0.52-0.70]), a lower LBR (29.81 vs. 43.95%, aOR=0.58 [0.49-0.67]), and a higher miscarriage rate (27.91 vs. 20.39%, aOR=1.40 [1.10-1.79]). The rates of biochemical pregnancy and ectopic pregnancy were both comparable between the two groups.

## Discussion

In this study, we found that a thin endometrium (EMT <8 mm) had adverse effects on perinatal outcomes, in terms of an earlier delivery, lower mean birth weight, and higher risks of PTB, LBW, and gestational diabetes. In addition, an EMT <8 mm was associated with lower rates of clinical pregnancy and live birth, and a higher rate of miscarriage.

Over the past decades, attention has been paid mainly to the effect of EMT on IVF success; however, previous studies have shown conflicting results. Some studies found lower rates of clinical pregnancy and live birth in patients with thin endometria, while others observed no significant effect of EMT on IVF success rates. Discrepant results among studies may be owing to several reasons. First, definitions for a thin endometrium vary among studies, and different cut-offs (such as 7, 7.5, and 8 mm) have been used. In this study, we used an EMT of 8 mm as the cut-off, based on our FET protocol in which the target EMT is ≥8 mm. Second, some studies that found no effect of EMT on pregnancy outcomes were of small sample size. These studies included only a few patients with thin endometria, and therefore might have had insufficient power to detect a significant difference. Third, multiple embryo transfer may confound the nature of the relationship of EMT and IVF success. Unfortunately, most studies were based on the practice of multiple embryo transfer or a combination of single and multiple embryo transfers. Our study included only those cycles with a single blastocyst transfer.

In this study, restricted cubic spline curves showed that the clinical pregnancy rate did not increase when EMT was beyond 8 mm, but showed a plateau. This result indicates that EMT of 8 mm is a cut-off with clinical value. When an endometrium reaches 8 mm, it is sufficient for implantation. Therefore, the chance of pregnancy does not increase after 8 mm. As for the live birth rate, it increased gradually with increasing EMT. Thicker endometria might provide a better condition for fetal development, and thus result in a higher chance for live birth.

In the current study, the predicting capacity of EMT on pregnancy was low (AUC=0.55). In another word, patients with a thin endometrium still had an acceptable chance of achieving a pregnancy (The CPRs were 41.98% for cycles with an EMT <8 mm, and 55.71% for those with an EMT ≥8 mm). Such results are consistent with two recently published meta-analyses (2, 10), which argue that cancelation of embryo transfer with an indication of a thin endometrium is not justified. However, it should be noted that these two meta-analyses did not account for the risk of miscarriage. Miscarriage has been traditionally associated with embryo factors. More recent studies suggest that the endometrium also plays a role in miscarriage (8, 19). Our study showed a significantly increased risk of miscarriage in cycles with an EMT <8 mm. Therefore, embryo transfer should be performed with some caution in patients with a thin endometrium, and they should be informed of the potential risk for miscarriage.

There is a growing focus on IVF safety, in terms of maternal and neonatal outcomes. Martel et al. found that neonates of women with an EMT <7 mm were born 2 weeks earlier than those with an EMT ≥7 mm (20). However, they did not find a significant difference in PTB between groups, possibly owing to the small number of patients included. Hu et al. included a large cohort, and observed a 1.8-fold increased risk of PTB in women with an EMT <8 mm (14). Similarly, our study found that women with an EMT <8 mm were at a 1.7-fold increased risk of PTB.

EMT has been found to be associated with adverse obstetric complications. Oron et al. reported a higher risk of overall obstetric complications in patients with an EMT <7.5 mm (21). This study included only 864 singleton deliveries, and only 92 of them had thin endometria; therefore, it was unable to show a difference of individual obstetric complications. Another study including 5251 singleton deliveries found a 6-fold increased risk of placenta previa in patients with an EMT <9 mm (22). Our study showed an EMT <8 mm was associated with a significantly higher incidence of gestational diabetes. Furthermore, incidences of hypertension disorders and placenta accreta/increta were higher in the patients with an EMT <8 mm.

EMT has been demonstrated to be correlated with birth weight (20, 23). Moreover, prior studies also found a lower mean birth weight and a higher risk of LBW in patients with an EMT <8 mm (13). Our results support these findings, indicating that thin endometria result in decreased birth weight, and this effect translates to increased cases of LBW, and correspondingly to decreased cases of LGA and macrosomia. Several studies report a higher risk of SGA in cycles with thin endometria (12, 15); however, we did not observe such a difference in SGA between the two groups in our study. Noteworthily, studies reporting a significant association between EMT and SGA were based on fresh cycles. In contrast, our study was based on frozen cycles. It has been reported that frozen embryo transfer may result in a 160 g increase in birth weight compared with fresh embryo transfer (24). A plausible explanation for this finding is that the practice of frozen embryo transfer might increase the birth weight of neonates, and this effect may reduce the incidence of SGA. Indeed, another recently published study on frozen cycles also found a neutral association between EMT and SGA (14).

The mechanisms underlying the association between a thin endometrium and adverse perinatal outcomes remains elusive. In a natural cycle, spiral arteries constrict after ovulation, which reduces blood flow and oxygen tension to the functional layer of the endometrium during implantation (25). For a thin endometrium, the site of implantation is close to the basal layer of the endometrium; oxygen tension in this region is high, which may compromise placentation and fetal development.

Another mechanism is the abnormal vascular remodeling of spiral arteries. A thin endometrium is characterized by poor vascular development and reduced blood supply to the placenta. Consequently, this may compromise placentation and fetal growth, and thus lead to adverse perinatal outcomes. Additionally, placental hypoperfusion may also lead to oxidative stress, which is deleterious to fetal growth.

The choice between continuing with or canceling an embryo transfer is a dilemma in patients with thin endometria. Although some studies have argued that canceling or deferring an embryo transfer only based on a low EMT is not justified; our results do not support this notion, owing to the higher risks of miscarriage, PTB, and LBW in women with an EMT of <8 mm. Therefore, attempting to achieve the deepest EMT possible is optimal. It should be explained to patients with thin endometria that, although they have an acceptable chance of achieving a clinical pregnancy, the potential risks of miscarriage and adverse perinatal outcomes are high. Deferring to another cycle with a thicker endometrium should be considered for these patients. If an embryo has been transferred and has achieved a pregnancy in a patient with a thin endometrium, additional prenatal care should be recommended.

There is an ongoing debate regarding which method of endometrial preparation is best. Our previous study showed that CPR and LBR were both higher after the AC method compared with NC (26). However, little is known regarding perinatal outcomes after different endometrial preparation methods. In this study, no significant differences were found in maternal and neonatal outcomes among the cycles with NC, AC, or DR+AC protocols (**Table S5**).

The strengths of this study include its large sample size. A total of 10098 cycles were analyzed. Furthermore, all cycles were performed with the protocol of frozen-thawed single blastocyst transfer, and cycles with monozygotic twin pregnancies were excluded. This uniform cohort was ideal for analysis because of the absence of endometrium-embryo asynchrony resulting from hyperstimulation in fresh cycles, and there was no confounding effect of multiple embryo transfer. In addition, it was a single-center study. Embryo culturing conditions and the measurement of EMT were stable during the study period.

There are some limitations to this study. First, it was retrospective in nature. Nevertheless, we meticulously included a homogenous study population. Multivariable models were also used to adjust for known confounders. Second, the two groups of EMT <8 and ≥8 mm were not balanced, owing to the infrequency of thin endometria. Although the overall sample size was large, only 8.7% (879 cycles) had an EMT <8 mm, and the number of live births were additionally small (262 live births). Therefore, it was difficult to draw a robust conclusion from the results of analysis on several rare perinatal parameters. Third, EMT measurement is subjective, and intra- and inter-observer variations might exist. However, EMT was measured strictly according to the standard operation protocol of ISO-9001 files at our center, and all measurements were completed by three experienced sonographers during the study period.

In conclusion, a thin endometrium may be associated with earlier delivery, lower birth weight, and higher risks of PTB, LBW, and gestational diabetes. Additionally, a thin endometrium may result in lower rates of clinical pregnancy and live birth, and a higher miscarriage rate. Further studies are needed to confirm our findings, and to elucidate the underlying mechanisms.

## Data availability statement

The datasets presented in this study can be found in online repositories. The names of the repository/repositories and accession number(s) can be found in the article/**Supplementary Material**.

## Ethics statement

The studies involving human participants were reviewed and approved by the Institutional Review Board (IRB) of Tongji Hospital. The patients/participants provided their written informed consent to participate in this study.

## Author contributions

YZ, XD, BX and JA were responsible for the study conception. All authors contributed to the study design. LJ supervised the study. YZ collected the data. YZ and XD analyzed the data. All authors contributed to the interpretation of data. YZ drafted the article. XD, BC, JD, BX, JA, and LJ revised the manuscript. All authors approved the submitted version.

## Acknowledgments

The authors thank all the staff at the Reproductive Medicine Center, Tongji Hospital, for their work and support. The authors thank the infertile couples who participated in this study. The revised manuscript has been edited by the Charlesworth Author Services (https://www.cwauthors.com).

## Conflict of interest

The authors declare that the research was conducted in the absence of any commercial or financial relationships that could be construed as a potential conflict of interest.

## Publisher’s note

All claims expressed in this article are solely those of the authors and do not necessarily represent those of their affiliated organizations, or those of the publisher, the editors and the reviewers. Any product that may be evaluated in this article, or claim that may be made by its manufacturer, is not guaranteed or endorsed by the publisher.

## Supplementary material

Supplementary Figure 1 Association analysis of EMT and clinical pregnancy/live birth. **(A)** Dose-response association between EMT and the odds of clinical pregnancy; **(B)** Dose-response association between EMT and the odds of live birth; **(C)** ROC curve for EMT in the prediction of clinical pregnancy; **(D)** ROC curve for EMT in the prediction of live birth; **(E)** Forest plot of clinical pregnancy for women with different EMT; **(F)** Forest plot of live birth for women with different EMT. OR, odds ratio; EMT, endometrial thickness; AUC, area under the curve; REF, reference.

## References

[1] Ruiz-Alonso M Valbuena D Gomez C Cuzzi J Simon C. Endometrial receptivity analysis (ERA): Data versus opinions. Hum Reprod Open (2021) 2021(2):hoab011. doi: 10.1093/hropen/hoab011 33880420 PMC8045472

[2] Craciunas L Gallos I Chu J Bourne T Quenby S Brosens JJ. Conventional and modern markers of endometrial receptivity: A systematic review and meta-analysis. Hum Reprod Update (2019) 25(2):202–23. doi: 10.1093/humupd/dmy044 30624659

[3] Lai CW Yung SS Ng EH. Endometrial vascularity is lower in pregnancies with pregnancy-induced hypertension or small-for-gestational-age fetus in live birth after *in-vitro* fertilization. Ultrasound obstetrics gynecology (2014) 44(4):455–60. doi: 10.1002/uog.13309 24452850

[4] Vaegter KK Lakic TG Olovsson M Berglund L Brodin T Holte J. Which factors are most predictive for live birth after *in vitro* fertilization and intracytoplasmic sperm injection (IVF/ICSI) treatments? analysis of 100 prospectively recorded variables in 8,400 IVF/ICSI single-embryo transfers. Fertil Steril (2017) 107(3):641–8. doi: 10.1016/j.fertnstert.2016.12.005 28108009

[5] Liu KE Hartman M Hartman A Luo ZC Mahutte N. The impact of a thin endometrial lining on fresh and frozen-thaw IVF outcomes: An analysis of over 40 000 embryo transfers. Hum Reprod (2018) 33(10):1883–8. doi: 10.1093/humrep/dey281 PMC6145412 30239738

[6] Onogi S Ezoe K Nishihara S Fukuda J Kobayashi T Kato K. Endometrial thickness on the day of the LH surge: An effective predictor of pregnancy outcomes after modified natural cycle-frozen blastocyst transfer. Hum Reprod Open (2020) 2020(4):hoaa060. doi: 10.1093/hropen/hoaa060 33511290 PMC7821991

[7] Gingold JA Lee JA Rodriguez-Purata J Whitehouse MC Sandler B Grunfeld L. Endometrial pattern, but not endometrial thickness, affects implantation rates in euploid embryo transfers. Fertil Steril (2015) 104(3):620–8. doi: 10.1016/j.fertnstert.2015.05.036 PMC4561002 26079695

[8] Simeonov M Sapir O Lande Y Ben-Haroush A Oron G Shlush E. The entire range of trigger-day endometrial thickness in fresh IVF cycles is independently correlated with live birth rate. Reprod BioMed Online (2020) 41(2):239–47. doi: 10.1016/j.rbmo.2020.04.008 32532669

[9] Shakerian B Turkgeldi E Yildiz S Keles I Ata B. Endometrial thickness is not predictive for live birth after embryo transfer, even without a cutoff. Fertil Steril (2021) 116(1):130–7. doi: 10.1016/j.fertnstert.2021.02.041 33812651

[10] Kasius A Smit JG Torrance HL Eijkemans MJ Mol BW Opmeer BC. Endometrial thickness and pregnancy rates after IVF: A systematic review and meta-analysis. Hum Reprod Update (2014) 20(4):530–41. doi: 10.1093/humupd/dmu011 24664156

[11] Declercq E Luke B Belanoff C Cabral H Diop H Gopal D. Perinatal outcomes associated with assisted reproductive technology: The Massachusetts outcomes study of assisted reproductive technologies (MOSART). Fertil Steril (2015) 103(4):888–95. doi: 10.1016/j.fertnstert.2014.12.119 PMC4385441 25660721

[12] Guo Z Xu X Zhang L Zhang L Yan L Ma J. Endometrial thickness is associated with incidence of small-for-gestational-age infants in fresh *in vitro* fertilization-intracytoplasmic sperm injection and embryo transfer cycles. Fertil Steril (2020) 113(4):745–52. doi: 10.1016/j.fertnstert.2019.12.014 32147172

[13] Zhang J Liu H Mao X Chen Q Si J Fan Y. Effect of endometrial thickness on birthweight in frozen embryo transfer cycles: An analysis including 6181 singleton newborns. Hum Reprod (2019) 34(9):1707–15. doi: 10.1093/humrep/dez103 31398256

[14] Hu KL Kawai A Hunt S Li W Li X Zhang R. Endometrial thickness in the prediction of neonatal adverse outcomes in frozen cycles for singleton pregnancies. Reprod BioMed Online (2021) 43:553–60. doi: 10.1016/j.rbmo.2021.04.014 34332902

[15] Liu X Wu H Fu X Li J Zhang M Yan J. Association between endometrial thickness and birth weight in fresh IVF/ICSI embryo transfers: A retrospective cohort study of 9273 singleton births. Reprod BioMed Online (2021) 43:1087–94. doi: 10.1016/j.rbmo.2021.08.021 34600855

[16] Ribeiro VC Santos-Ribeiro S De Munck N Drakopoulos P Polyzos NP Schutyser V. Should we continue to measure endometrial thickness in modern-day medicine? The effect on live birth rates and birth weight. Reprod BioMed Online (2018) 36(4):416–26. doi: 10.1016/j.rbmo.2017.12.016 29361452

[17] Christopoulos G Vlismas A Salim R Islam R Trew G Lavery S. Fibroids that do not distort the uterine cavity and IVF success rates: An observational study using extensive matching criteria. BJOG (2017) 124(4):615–21. doi: 10.1111/1471-0528.14362 27921379

[18] Ai J Jin L Zheng Y Yang P Huang B Dong X. The morphology of inner cell mass is the strongest predictor of live birth after a frozen-thawed single embryo transfer. Front Endocrinol (Lausanne) (2021) 12:621221. doi: 10.3389/fendo.2021.621221 33716973 PMC7943864

[19] Gallos ID Khairy M Chu J Rajkhowa M Tobias A Campbell A. Optimal endometrial thickness to maximize live births and minimize pregnancy losses: Analysis of 25,767 fresh embryo transfers. Reprod BioMed Online (2018) 37(5):542–8. doi: 10.1016/j.rbmo.2018.08.025 30366837

[20] Martel RA Blakemore JK Grifo JA. The effect of endometrial thickness on live birth outcomes in women undergoing hormone-replaced frozen embryo transfer. F S Rep (2021) 2(2):150–5. doi: 10.1016/j.xfre.2021.04.002 PMC8267379 34278346

[21] Oron G Hiersch L Rona S Prag-Rosenberg R Sapir O Tuttnauer-Hamburger M. Endometrial thickness of less than 7.5 mm is associated with obstetric complications in fresh IVF cycles: a retrospective cohort study. Reprod BioMed Online (2018) 37(3):341–8. doi: 10.1016/j.rbmo.2018.05.013 30146441

[22] Jing S Li X Zhang S Gong F Lu G Lin G. The risk of placenta previa and cesarean section associated with a thin endometrial thickness: A retrospective study of 5251 singleton births during frozen embryo transfer in China. Arch Gynecol Obstet (2019) 300(5):1227–37. doi: 10.1007/s00404-019-05295-6 31552485

[23] Moffat R Beutler S Schötzau A De Geyter M De Geyter C. Endometrial thickness influences neonatal birth weight in pregnancies with obstetric complications achieved after fresh IVF-ICSI cycles. Arch Gynecol Obstet (2017) 296(1):115–22. doi: 10.1007/s00404-017-4411-z 28589476

[24] Chen ZJ Shi Y Sun Y Zhang B Liang X Cao Y. Fresh versus frozen embryos for infertility in the polycystic ovary syndrome. N Engl J Med (2016) 375(6):523–33. doi: 10.1056/NEJMoa1513873 27509101

[25] Casper RF. It's time to pay attention to the endometrium. Fertil Steril (2011) 96(3):519–21. doi: 10.1016/j.fertnstert.2011.07.1096 21880272

[26] Zheng Y Dong X Huang B Zhang H Ai J. The artificial cycle method improves the pregnancy outcome in frozen-thawed embryo transfer: a retrospective cohort study. Gynecol Endocrinol (2015) 31(1):70–4. doi: 10.3109/09513590.2014.958988 25223893

