# Supplementary figures and images for: Thin endometrium is associated with higher risks of preterm birth and low birth weight after frozen single blastocyst transfer
Source: Front Endocrinol (Lausanne). 2022 Nov 10;13:1040140. doi: 10.3389/fendo.2022.1040140 (PMC9685422; doi:10.3389/fendo.2022.1040140)

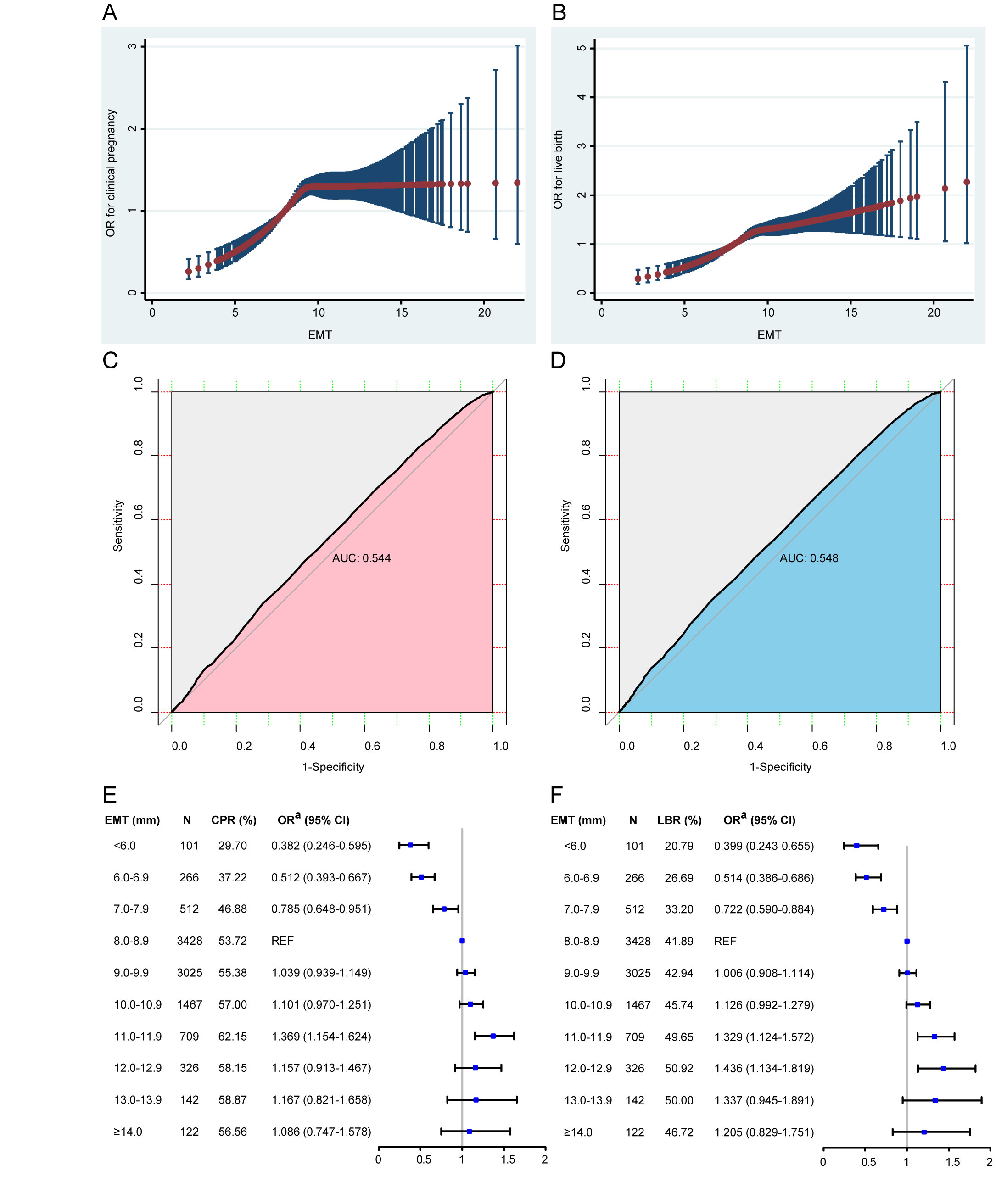

Supplement: Supplementary Figure 1 — Association analysis of EMT and clinical pregnancy/live birth. (A) Dose-response association between EMT and the odds of clinical pregnancy; (B) Dose-response association between EMT and the odds of live birth; (C) ROC curve for EMT in the prediction of clinical pregnancy; (D) ROC curve for EMT in the prediction of live birth; (E) Forest plot of clinical pregnancy for women with different EMT; (F) Forest plot of live birth for women with different EMT. OR, odds ratio; EMT, endometrial thickness; AUC, area under the curve; REF, reference. [file Image_1.jpeg]
