# Supplementary material for: Thin endometrium is associated with higher risks of preterm birth and low birth weight after frozen single blastocyst transfer
Source: Front Endocrinol (Lausanne). 2022 Nov 10;13:1040140. doi: 10.3389/fendo.2022.1040140 (PMC9685422; doi:10.3389/fendo.2022.1040140)
Supplement: Supplementary file 2 [file Table_1.docx]

**Table S1 Univariable analysis of live birth**

|  | Live birth | Not live birth | P value |
| --- | --- | --- | --- |
| No. of cycles | 4314 | 5784 |  |
| Age (year) | 30.95 ± 4.09 | 32.62 ± 4.91 | <0.0001 |
| BMI (kg/m^2^) | 21.79 ± 3.00 | 21.85 ± 2.98 | 0.3124 |
| Primary infertility, n (%) | 1640 (38.02) | 2541 (43.93) | <0.0001 |
| Duration of infertility (year) | 3.26 ± 2.31 | 3.58 ± 2.89 | <0.0001 |
| Infertility cause, n (%)  Tubal factors  Endometriosis  Male factors  Ovulation disorders  Unexplained factor  Diminished ovarian reserve  Others | 1497 (34.70)  274 (6.35)  570 (13.21)  897 (20.79)  217 (5.03)  750 (17.39)  109 (2.53) | 2206 (38.14)  414 (7.16)  430 (7.43)  1202 (20.78)  130 (2.25)  1218 (21.06)  184 (3.18) | <0.0001 |
| AMH (ng/ml) | 5.40 ± 2.04 | 3.98 ± 3,23 | <0.0001 |
| AFC | 13.44 ± 6.07 | 11.97 ± 5.90 | <0.0001 |
| No. of oocytes | 11.39 ± 4.56 | 10.72 ± 4.86 | <0.0001 |
| Fertilization method, n (%)  IVF  ICSI | 3085 (71.51)  1229 (28.49) | 4085 (70.63)  1699 (29.37) | 0.3320 |
| No. of available embryos | 4.17 ± 2.26 | 3.16 ± 2.81 | <0.0001 |
| Endometrial preparation, n (%)  Natural cycle  Artificial cycle  Down-regulation + artificial cycle | 228 (5.29)  3649 (84.59)  437 (10.13) | 334 (5.77)  4878 (84.34)  572 (9.89) | 0.5421 |
| Endometrial thickness  Mean (mm)  Cut-off, n (%)  <8 mm  ≥ 8 mm | 9.49 ± 1.53  262 (6.07)  4052 (93.93) | 9.22 ± 1.55  617 (10.67)  5167 (89.33) | <0.0001  <0.0001 |
| Day of blastocyst development, n (%)  Day 5  Day 6 | 2640 (61.20)  1674 (38.80) | 2729 (47.18)  3055 (52.82) | <0.0001 |
| Blastocyst quality, n (%)  Good  Fair  Poor | 561 (13.00)  2729 (63.26)  1024 (23.74) | 423 (7.31)  3170 (54.81)  2191 (37.88) | <0.0001 |
| Blastocyst stage, n (%)  3  4  5  6 | 847 (19.63)  3230 (74.87)  143 (3.31)  94 (2.18) | 1467 (25.36)  3936 (68.05)  217 (3.75)  164 (2.84) | <0.0001 |
| ICM score, n (%)  A  B  C | 574 (13.31)  3716 (86.14)  24 (0.56) | 440 (7.61)  5280 (91.29)  64 (1.11) | <0.0001 |
| TE score, n (%)  A  B  C | 421 (9.76)  2875 (66.64)  1018 (23.60) | 346 (5.98)  3291 (56.90)  2147 (37.12) | <0.0001 |
