# Supplementary material for: Thin endometrium is associated with higher risks of preterm birth and low birth weight after frozen single blastocyst transfer
Source: Front Endocrinol (Lausanne). 2022 Nov 10;13:1040140. doi: 10.3389/fendo.2022.1040140 (PMC9685422; doi:10.3389/fendo.2022.1040140)
Supplement: Supplementary file 3 [file Table_2.docx]

**Table S2 RCS curves**

The p25%, median, P75% and P95% of EMT are as follow:

| Freq. | P25 (EMT) | Med (EMT) | P75 (EMT) | P95 (EMT) |
| --- | --- | --- | --- | --- |
| 10098 | 8.4 | 9.1 | 10 | 12.1 |

Then cubic variates 1-3 were generated.

Multivariable logistic regression was used to analyze the associations between EMT and live birth, with age, BMI, type of infertility, duration of infertility, IVF indication, the number of oocytes retrieved, blastocyst stage, and blastocyst scores as covariates. The details are as follow:

|  | OR | 95% CI | P value |
| --- | --- | --- | --- |
| Age | 0.927 | 0.919-0.936 | <0.0001 |
| BMI | 1.009 | 0.995-1.023 | 0.1911 |
| Infertility type  Primary  Secondary | REF  1.228 | REF  0.727-2.119 | 0.6425 |
| Duration of infertility | 0.791 | 0.369-1.695 | 0.7686 |
| IVF indication |  |  |  |
| EMT  <8 mm  ≥8 mm | 0.577  REF | 0.494-0.674  REF | <0.0001 |
| No. of oocytes retrieved | 1.032 | 0.982-1.083 | 0.3521 |
| Blastocyst stage  3  4  5  6 | REF  1.294  1.129  0.952 | REF  1.171-1.431  0.893-1.428  0.724-1.252 | -  0.0003  0.6652  0.2041 |
| ICM score  A  B  C | 2.992  2.161  REF | 1.819-4.921  1.340-3.483  REF | <0.0001  0.0085  - |
| TE score  A  B  C | 1.864  1.763  REF | 1.543-2.252  1.607-1.934  REF | 0.0002  <0.0001  - |

Then RCS curves were generated, in the range of 2.2-22 mm, and the reference was set as “8 mm”
