# Supplementary material for: Thin endometrium is associated with higher risks of preterm birth and low birth weight after frozen single blastocyst transfer
Source: Front Endocrinol (Lausanne). 2022 Nov 10;13:1040140. doi: 10.3389/fendo.2022.1040140 (PMC9685422; doi:10.3389/fendo.2022.1040140)
Supplement: Supplementary file 4 [file Table_3.docx]

**Table S3 Association between EMT and cycle outcomes**

|  | EMT <8 mm | EMT ≥8 mm | P value |
| --- | --- | --- | --- |
| No of cycles | 879 | 9219 |  |
| Biochemical pregnancy rate, n (%)  OR^a^ (95% CI) | 72 (8.19)  1.193 (0.924-1.541) | 646 (7.01)  REF | 0.1919  0.1761 |
| CPR, n (%)  OR^a^ (95% CI) | 369 (41.98)  0.602 (0.521-0.695) | 5136 (55.71)  REF | <0.0001  <0.0001 |
| LBR, n (%)  OR^a^ (95% CI) | 262 (29.81)  0.577 (0.494-0.674) | 4052 (43.95)  REF | <0.0001  <0.0001 |
| Miscarriage rate per CP, n (%)  OR^a^ (95% CI) | 103 (27.91)  1.404 (1.101-1.790) | 1047 (20.39)  REF | 0.0006  0.0063 |
| Ectopic pregnancy rate per CP, n (%)  OR^a^ (95% CI) | 4 (1.08)  1.445 (0.509-4.102) | 37 (0.72)  REF | 0.4326  0.4896 |

Note:

^a^: adjustment with multivariate logistic regression model; OR: odds ratio; CPR: clinical pregnancy rate; LBR: live birth rate; REF: reference
