# Supplementary material for: Thin endometrium is associated with higher risks of preterm birth and low birth weight after frozen single blastocyst transfer
Source: Front Endocrinol (Lausanne). 2022 Nov 10;13:1040140. doi: 10.3389/fendo.2022.1040140 (PMC9685422; doi:10.3389/fendo.2022.1040140)
Supplement: Supplementary file 5 [file Table_4.docx]

**Table S4**

**Independent covariates in the multivariable logistic regression model for preterm birth**

|  | β’ | OR | 95% CI | P value |
| --- | --- | --- | --- | --- |
| Age | 0.1110 | 1.051 | 1.026-1.076 | <0.0001 |
| BMI | 0.1157 | 1.073 | 1.039-1.107 | <0.0001 |
| Endometrial thickness  <8 mm  ≥8 mm | 0.0690  - | 1.694  REF | 1.185-2.424  - | 0.0039  - |
| Infertility type  Primary  Secondary | -  0.0822 | REF  1.694 | -  1.228-2.339 | -  0.0013 |
| TE score  A  B  C | 0.0454  -0.0977  - | 1.088  0.762  REF | 0.708-1.671  0.604-0.961  - | 0.2726  0.0099  - |

**Independent covariates in the multivariable logistic regression model for low birth weight**

|  | β’ | OR | 95% CI | P value |
| --- | --- | --- | --- | --- |
| Age | 0.0962 | 1.044 | 1.007-1.081 | 0.0178 |
| Endometrial thickness  <8 mm  ≥8 mm | 0.0934  - | 2.048  REF | 1.269-3.304  - | 0.0033  - |
