# Supplementary material for: Thin endometrium is associated with higher risks of preterm birth and low birth weight after frozen single blastocyst transfer
Source: Front Endocrinol (Lausanne). 2022 Nov 10;13:1040140. doi: 10.3389/fendo.2022.1040140 (PMC9685422; doi:10.3389/fendo.2022.1040140)
Supplement: Supplementary file 6 [file Table_5.docx]

**Table S5 Perinatal outcomes according to endometrial preparation method**

|  | **NC** | **AC**  **OR^a^ (95% Cl)** | **DR+AC**  **OR^a^ (95% Cl)** |
| --- | --- | --- | --- |
| No. of deliveries | 562 | 8527 | 1009 |
| Preterm birth, n (%) | REF | 1.021 (0.650-1.603) | 1.177 (0.693-1.997) |
| Very preterm birth, n (%) | REF | 1.336 (0.320-5.580) | 1.082 (0.320-5.580) |
| Gestational diabetes, n (%) | REF | 1.063 (0.549-2.059) | 0.762 (0.334-1.737) |
| Hypertension, n (%) | REF | 1.058 (0.455-2.456) | 0.640 (0.218-1.879) |
| Placenta previa, n (%) | REF | 0.894 (0.427-1.873) | 0.958 (0.393-2.334) |
| Placenta abruption, n (%) | NA | NA | NA |
| Placenta accreta/increta, n (%) | REF | 0.702 (0.162-3.051) | 0.804 (0.132-4.890) |
| PROM, n (%) | REF | 1.118 (0.148-8.433) | 1.953 (0.216-17.649) |
| LBW, n (%) | REF | 0.933 (0.495-1.759) | 1.098 (0.519-2.325) |
| VLBW, n (%) | REF | 1.429 (0.191-10.677) | 1.094 (0.098-12.191) |
| SGA, n (%) | REF | 1.224 (0.616-2.431) | 0.878 (0.383-2.009) |
| LGA, n (%) | REF | 0.991 (0.675-1.455) | 1.134 (0.726-1.772) |
| Macrosomia, n (%) | REF | 1.183 (0.708-1.977) | 1.433 (0.796-2.580) |
| Congenital anomaly, n (%) | REF | 1.407 (0.181-10.917) | 2.426 (0.258-22.830) |
| PICU admission, n (%) | NA | NA | NA |
| Perinatal death, n (%) | NA | NA | NA |
